# Proteomic analysis of the regulatory networks of ClpX in a model cyanobacterium *Synechocystis* sp. PCC 6803

**DOI:** 10.3389/fpls.2022.994056

**Published:** 2022-09-29

**Authors:** Yumeng Zhang, Yaqi Wang, Wei Wei, Min Wang, Shuzhao Jia, Mingkun Yang, Feng Ge

**Affiliations:** ^1^ State Key Laboratory of Freshwater Ecology and Biotechnology, Institute of Hydrobiology, Chinese Academy of Sciences, Wuhan, China; ^2^ Key Laboratory of Algal Biology, Institute of Hydrobiology, Chinese Academy of Sciences, Wuhan, China; ^3^ College of Advanced Agricultural Sciences, University of Chinese Academy of Sciences, Beijing, China; ^4^ The Analysis and Testing Center, Institute of Hydrobiology, Chinese Academy of Sciences, Wuhan, China

**Keywords:** cyanobacteria, proteases, proteostasis, quantitative proteomics, ClpX

## Abstract

Protein homeostasis is tightly regulated by protein quality control systems such as chaperones and proteases. In cyanobacteria, the ClpXP proteolytic complex is regarded as a representative proteolytic system and consists of a hexameric ATPase ClpX and a tetradecameric peptidase ClpP. However, the functions and molecular mechanisms of ClpX in cyanobacteria remain unclear. This study aimed to decipher the unique contributions and regulatory networks of ClpX in the model cyanobacterium *Synechocystis* sp. PCC 6803 (hereafter *Synechocystis*). We showed that the interruption of *clpX* led to slower growth, decreased high light tolerance, and impaired photosynthetic cyclic electron transfer. A quantitative proteomic strategy was employed to globally identify ClpX-regulated proteins in *Synechocystis* cells. In total, we identified 172 differentially expressed proteins (DEPs) upon the interruption of *clpX*. Functional analysis revealed that these DEPs are involved in diverse biological processes, including glycolysis, nitrogen assimilation, photosynthetic electron transport, ATP-binding cassette (ABC) transporters, and two-component signal transduction. The expression of 24 DEPs was confirmed by parallel reaction monitoring (PRM) analysis. In particular, many hypothetical or unknown proteins were found to be regulated by ClpX, providing new candidates for future functional studies on ClpX. Together, our study provides a comprehensive ClpX-regulated protein network, and the results serve as an important resource for understanding protein quality control systems in cyanobacteria.

## Introduction

Cyanobacteria are a large group of prokaryotic photoautotrophic microorganisms that play crucial roles in the global carbon and nitrogen cycles (Khalifa et al., 2021). As one of the oldest forms of life on Earth, cyanobacteria are present in almost every habitat, including various extreme environments and adverse physiological growth conditions (Schirrmeister et al., 2011). Extreme environments often disturb cellular protein homeostasis (proteostasis), resulting in protein denaturation and oxidative damage, which leads to cell death. To cope with harsh and changing environmental conditions, cyanobacteria have evolved versatile protein quality control (PQC) mechanisms to sense environmental signals and implement adaptive changes (Franklin, 2021; Cui et al., 2021).

In cyanobacteria, unlike the ubiquitin-proteasome system found in eukaryotic systems, proteostasis is regulated by the PQC system, mainly depending on AAA+ (ATPases associated with a variety of cellular activities) proteolytic machines, which are composed of two distinct parts: molecular chaperones and proteases (He and Mi, 2016). Among the diverse proteolytic machines, including ClpXP, ClpCP, and HslUV, the ClpXP proteolytic complex is the representative AAA+ proteolytic machine (Olivares et al., 2016) and is regarded as the most characterized and conserved (Baker and Sauer, 2012; Stahlhut et al., 2017). It consists of a ring hexamer of the ClpX subunit (Glynn et al., 2009) and the self-compartmentalized serine protease ClpP, in which two stacked heptameric rings enclose a barrel-shaped chamber (Stanne et al., 2007; Alexopoulos et al., 2012).

ClpX is a hexameric ATPase with diverse functions, including substrate binding, adaptor functions, protein unfolding, and polypeptide translocation. Unfolding and translocation require ATP binding and hydrolysis to power changes in enzyme conformation that drive these mechanical processes (Kim et al., 2000; Joshi et al., 2004). At the same time, ClpP needs to bind to ClpX to cleave polypeptides that are translocated into its proteolytic chamber. The resulting peptide fragments must be small enough to exit the chamber and subsequently be degraded by exopeptidases to free amino acids (Baker and Sauer, 2012).

The ClpXP proteolytic complex has been revealed to participate in the modulation of several cellular activities, including cell division (Camberg et al., 2011), cell cycle regulation (Lau et al., 2015), and bacterial virulence (Li et al., 2010), by precisely degrading multiple regulatory proteins. Nevertheless, independent of ClpP, ClpX is known to prevent protein aggregation (Burton and Baker, 2005), disassemble preformed aggregates (Burton and Baker, 2005), and unfold proteins without degradation (Baker and Sauer, 2012). Interestingly, some organisms such as yeast do not contain ClpP but only ClpX (Whitman et al., 2018). ClpX influences the transcription of genes involved in peptidoglycan synthesis, cell division, and the type seven secretion system in *Staphylococcus aureus* (Jensen et al., 2019). Furthermore, ClpX has been reported to be an important factor in subverting host immune clearance mechanisms in *Bacillus anthracis* (McGillivray et al., 2009) and is engaged in McpA proteolysis, which is modulated by the cell cycle in *Caulobacter crescentus* (Tsai and Alley, 2001).

As the molecular chaperone, ClpX is required for substrate recognition and delivery of target proteins to the ClpP peptidase chamber, suggesting that ClpX functions in a manner that contributes to the degradation of the ClpXP proteolytic complex, and thus precisely regulates multiple cellular processes. In cyanobacteria, a study revealed that ClpX is required to regulate the circadian gating of cell division (Cohen et al., 2018). In addition, *clpX* can affect the circadian period by regulating the transcription of ribosomal protein genes in cyanobacteria (Imai et al., 2013). However, our understanding of the functions and molecular mechanisms of ClpX in cyanobacteria is still unknown.

Hence, we aimed to investigate the unique contributions and regulatory network of ClpX in a model cyanobacterium *Synechocystis* sp. PCC 6803 (hereafter *Synechocystis*). *Synechocystis* is one of the most widely used model organisms for photosynthesis and carbon metabolism studies (Knoop et al., 2010), and is amenable to genetic modification (Yu et al., 2013). In this study, we constructed a *clpX* insertion mutant of *Synechocystis* and found that depletion of *clpX* results in slower growth, decreased high light tolerance, and impaired cyclic photosynthetic electron transfer. We then used a quantitative proteomic strategy to identify the ClpX-regulated proteins in *Synechocystis*. Based on the results of the proteomic and functional studies, we constructed a ClpX regulatory network in *Synechocystis* and provided novel insights into the functions and molecular mechanisms of the PQC system in cyanobacteria.

## Materials and methods

### Cyanobacteria strains and culture conditions

*Synechocystis* cells were photoautotrophically cultured in liquid BG11 medium under constant illumination of 40 μmol photons m^−2^ s ^−1^, aerated with filtered air at 30°C. To determine growth rates, the OD_730_ was measured every 12 h using a spectrophotometer (MAPADA, Shanghai, China). The growth curve under high light was measured under constant illumination of 250 μmol photons m^−2^ s ^−1^ for a total of 96h. For high light treatments, logarithmic growth phase cells (OD_730_ = 0.7 to 0.8) were immediately illuminated at 250 μmol photons m^−2^ s ^−1^ for 1.5 h.

### Mutant construction

The *clpX* mutant strain was constructed by homologous recombination. Briefly, an approximately 500 bp sequence flanking the *clpX* gene derived from *Synechocystis* genomic DNA was amplified by PCR using the following primers: *clpX*-AF/*clpX*-AR and *clpX*-BF/*clpX*-BR. The kanamycin resistance cassette (derived from the PRL446 plasmid) was amplified using a pair of primers, *kana*-F, and *kana*-R. The PCR products were then fused by fusion PCR. The fused fragment was then inserted into the TA cloning vector PMD19-T (Takara, Japan), and subsequently transformed into *Synechocystis* strain as previously described (Carpentier, 2004). In a word, the *clpX* gene was inactivated by insertion of a kanamycin resistance cassette, and Δ*clpX* was regarded as *clpX* insertion mutant. The transformants were selected on BG11 medium supplemented with kanamycin (50μg/ml) and further confirmed by PCR using the primers *clpX*-F and *clpX*-R. All the primers and conditions for the PCR amplification are listed in **Table S1**.

### Photosynthetic oxygen evolution

For photosynthetic oxygen evolution rate assay, cells in the exponential growth phase were harvested and adjusted to a final concentration of 1.0 (OD_730_) with fresh BG11 liquid media. *Synechocystis* strains were determined under cell culture conditions using an oxygen electrode system (Hansatech Instruments Ltd, Norfolk, UK) while maintaining a 30°C circulating water bath and stirring the cell suspension, as previously described (Nomura et al., 2006). The saturating light intensity was measured at 800 μE m^–2^ s ^–1^, and 10 mM NaHCO_3_ was added as the electron acceptor.

### Chlorophyll fluorescence

For chlorophyll fluorescence measurement, cells in the exponential growth phase were harvested and adjusted to a final concentration of 0.8 (OD_730_) with fresh BG11 liquid media. After dark adaptation for 15 min, cell viability was determined using a Dual-PAM-100 fluorescence photosynthesis analyzer (Heinz Walz Gmbh, Effeltrich, Germany) at room temperature. The minimum fluorescence (*F_0_*) and maximum fluorescence (*F_m_*) levels were detected using the Dual-PAM software, and the maximal photochemical efficiency of photosystem II (PSII) in the dark-adapted state was calculated using the following formula: *F_v_*/*F_m_* = (*F_m_*−*F_o_*)/*F_m_* as previously described (Campbell et al., 1998). Transient increases in chlorophyll fluorescence after turning off actinic light (AL) were monitored as previously described (Shikanai et al., 1998).

### P700^+^ oxidation-reduction kinetics analysis

For P700^+^ oxidation-reduction kinetics, cells in the exponential growth phase were harvested and adjusted to a final concentration of 3.0 (OD_730_). After dark adaptation for 15 min, the redox state of P700^+^ was measured using Dual-PAM-100 under an absorbance signal from 820 nm to 860 nm in the absence of 10 mM dichlorophenyl dimethylurea (DCMU), and the reduction kinetics were fitted and calculated using GraphPad Prism software (version 8.4) (https://www.graphpad.com/updates) (Swift and Sciences, 1997), as previously described (Zhao et al., 1998).

### Bright-field and fluorescence microscopy

*Synechocystis* cells were collected by centrifugation at 5,000 g for 5 min at 30°C and washed three times with phosphate-buffered saline (PBS). Then visualized using a fluorescent microscope (Olympus, BX53, Japan) in the bright-field and RFP channel at a magnification of 100×.

### Transmission electron microscopy

Samples were prepared as previously described (Golecki, 1988). Briefly, *Synechocystis* cells were collected by centrifugation at 5,000 g for 5 min at 30°C, fixed overnight with 2.5% (v/v) glutardialdehyde, incubated in 1% (v/v) osmium tetroxide at 4°C for 16h and dehydrated through a graded ethanol series (Mohr et al., 2010). Then the samples were embedded in 1% Seakem agarose and cut into ultrathin sections. Ultrathin sections were mounted on pioloform-coated copper grids and poststained with 2% uranyl acetate and lead citrate (Reynolds, 1963). Micrographs were recorded using a transmission electron microscope system (Hitachi, HT-7700, Japan) at 80 kV.

### Whole-cell absorption spectra

*Synechocystis* cells were harvested at the mid-exponential growth phase and concentrated to the same optical density at 750 nm. Absorbance was detected using a SpectraMax M5 platform (Molecular Devices, America). Excitation was at 488 nm, and emission was collected from 400 to 750 nm with a sampling interval of 2 nm. Each spectrum is the average of three measurements.

### Measurement of intracellular ROS

The intracellular ROS production was assessed by using ROS assay kit ((Beyotime, China) according to the user’s manual protocols. Briefly, *Synechocystis* cells were collected and incubated with PBS buffer containing 10µM 6-carboxy-2’,7’-dichlorodihydrofluorescein diacetate (DCFH-DA) and 10µg/mL Rosup for 30 min (Zhu et al., 2020). The fluorescence signal of ROS was detected by the SpectraMax M5 platform (Molecular Devices, America) at the emission wavelength of 525 nm and excitation wavelength of 488 nm.

### Lipid peroxidation assay

The Lipid peroxidation was assessed by using MDA assay kit (Beyotime, China) according to the user’s manual protocols. *Synechocystis* cells were harvested and lysed by sonication (Scientz Biotechnology, China) for 20 min at 135 W, on ice. Supernatants were collected by centrifugation at 5,000 g for 20 min at 4°C and quantified using the BCA Protein Assay Kit (Beyotime, China). Supernatants were then mixed with the same volume of 20% (w/v) trichloroacetic acid (TCA) containing 0.65% (w/v) thiobarbituric acid (TBA). After incubation at 100°C for 15 min, the mixed samples were centrifuged at 1,000×g for 10 min, and the absorbance of the supernatant at 532 nm was measured by the SpectraMax M5 platform (Molecular Devices, America). For quantification, the range of the MDA standard curve was from 1 to 25 μmol.

### Protein extraction and trypsin digestion

Samples for proteomic analysis were harvested at the mid-exponential growth phase by centrifuging at 5,000 g for 5 min at 4°C and washed three times with phosphate-buffered saline (PBS). WT and Δ*clpX* samples were from three independent cell cultures. The samples were further resuspended in pre-cooled lysis buffer (PBS supplemented with 1 mM phenylmethylsulfonyl fluoride) and lysed by sonication (Scientz Biotechnology, Ningbo, China) for 20 min at 135 W, on ice. Undissolved cellular debris was removed by centrifugation at 5,000 g for 20 min at 4°C, and the supernatant was collected and quantified using the BCA Protein Assay Kit (Beyotime, Jiangsu, China). 100 μg of each sample was reduced by 25 mM dithiothreitol (DTT) at 37°C for 1 h and alkylated using 50 mM iodoacetamide (IAM) for 1 h in the dark to block reduced cysteine residues. The samples were digested with trypsin (1:100 w/w) for 6 h, and then additional trypsin (1:100 w/w) was added for a total of 24h digestion at 37°C. Following this, 0.1% (v/v) trifluoroacetic acid (TFA) was added to the resulting peptide mixtures to terminate the reaction. Samples were desalted using self-packed C18 SPE columns (C18, SBEQ-CA0801, Anple, shanghai) and dried using a vacuum centrifuge.

### Tandem mass tag labeling and high-performance liquid chromatography fractionation

Peptide samples were labeled using the TMT reagent 6-Plex kit (Thermo Fisher Scientific), according to the manufacturer’s instructions. The samples were labeled as follows: WT1:127N; WT2:128C; WT1:130N; ClpX-1:127C; ClpX-2:129N; ClpX-3:130C. The labeled peptides were mixed in equal amounts, resuspended in 5 mM NH_4_OH, and fractionated using an LC20AD high-pressure pump (Shimadzu Corporation, Kyoto, Japan). In detail, peptides were loaded onto a Waters XBridge Shield C18 RP column (4.6 mm * 250 mm, 3.5 μm particle size) and washed with a gradient of buffer B (5 mM NH_4_OH in 80% acetonitrile) 5% to 80% at a rate of 1 mL/min for 90 min. Finally, the eluted peptides were combined into 12 fractions and dried using vacuum centrifugation.

### LC-MS/MS analysis

Peptides were dissolved in 1% formic acid and loaded onto an analytical column (C18, 75 µm × 50 cm, 2 μm, Thermo Fisher Scientific) using an EASY-nLC 1200 System (Thermo Fisher Scientific). The peptides were eluted using solvent B (0.1% formic acid in 80% ACN, v/v) at a constant flow rate of 250 nl/min with a linear solvent gradient: 0−6 min, 2−10% B; 6−51 min, 10−20% B; 51−58 min, 20−80% B; 58−62 min, 80% B; 62−63 min, 80−2% B; 63−70 min, 2% B. Subsequently, 2μg of each sample was injected into a nano electrospray ion source, ionized and sprayed into a Q Exactive HF-X mass spectrometer (Thermo Fisher Scientific). MS data collection was performed using Xcalibur 3.0, in data-dependent acquisition mode with automatic alteration (1MS scan followed by 20MS/MS scans). A full MS scan with an m/z range of 350–1800 was acquired at a 60,000 resolution with a minimum signal intensity of 10,000. The top 15 precursor ions with charge states of 2–6 were selected for MS/MS fragmentation by high-energy collision dissociation (HCD) with a normalized collision energy of 25%. The electrospray voltage was set at 2.2 kV. The dynamic exclusion duration of the precursor ion was set to 30 s, and the isolation width of the precursor ion was set to 1.4 m/z. The maximum injection times were 20 ms and 200 ms for MS and MS/MS, respectively.

### Protein identification and quantification

All MS/MS spectra were searched against the *Synechocystis* protein database from the CyanoBase online website (http://genome.kazusa.or.jp/cyanobase, released in 2015) (Fujisawa et al., 2017) combined with the reverse decoy database and common contaminants using MaxQuant software (version 1.6.15.0) (https://www.maxquant.org/) (Cox and Mann, 2008). The parameters were set as follows: two maximum missed cleavage sites were permitted for trypsin; carbamidomethylation (Cys) was set as a fixed modification; oxidation (Met), deamidation (Asn/Gln), and acetylation (protein N-terminal) were set as variable modifications. 6-PlexTMT was set as a reporter ion. The mass deviations of precursor ions and fragment ions were set to 20 ppm and 0.02 Da, respectively, and the false discovery rate (FDR) thresholds for peptide and protein identification were specified at a maximum of 1%. Peptide sequences with less than six amino acids were excluded, and proteins that exceeded one unique peptide were identified. Quantification was performed using the Perseus software (version 1.5.6.0) (https://maxquant.net/perseus/) and Microsoft Excel (version Home & Student 2021). Only the proteins identified and quantified in three biological replicates were used for relative quantification. A two-sample Student’s t-test was used for the statistical evaluation. Differentially expressed proteins were defined as fold-change ≥ 1.2, ≤ 0.83, and p < 0.05.

### Bioinformatics analysis

To analyze the biological functions of the identified differentially expressed proteins (DEPs), they were grouped into biological process, molecular function, and cellular component classes based on the gene ontology (GO) terms using Blast2GO software (https://www.blast2go.com/) (Conesa and Gotz, 2008). Enrichment analyses of GO terms were performed using the DAVID bioinformatics resource (https://david.ncifcrf.gov/) (Huang et al., 2009). For our data, the corresponding *p*-value < 0.05 was considered statistically significant. The subcellular localization of the bacterial proteins was predicted using PSORTb (version3.0) (https://www.psort.org/psortb/) (Yu et al., 2010). To further explore the function of the DEPs, they were mapped to metabolic pathways using the KEGG database (https://www.kegg.jp/). The interaction network of DEPs was predicted by the STRING database (https://cn.string-db.org/) (Mering et al., 2003), further classified using the Markov Cluster Algorithm (MCL) clustering option, and visualized using Cytoscape (version3.8.0) (https://cytoscape.org/) (Shannon et al., 2003). Protein homologies were analyzed using BLASTP in the NCBI database. The domains of the Clp protease family in *Synechocystis* were annotated using the SMART online website (http://smart.embl-heidelberg.de/) (Schultz et al., 2000) and visualized using the IBS software (version1.0.3). The conserved motifs of ClpX proteins were predicted using the MEME online suite (http://meme-suite.org/tools/meme) with default settings (Bailey et al., 2009). To investigate the evolutionary conservation of the ClpX protein, a phylogenetic tree was constructed using MEGA software (version 11) (https://megasoftware.net/) with the neighbor-joining algorithm (NJ) method (Kumar et al., 2018). To visualize the distribution of the Clp protease family, a genomic map was created by using the DNA plotter tool (https://www.sanger.ac.uk/tool/dnaplotter/) (Carver et al., 2009). The complete genome of *Synechocystis* was obtained from the NCBI for Biotechnology Information website (https://www.ncbi.nlm.nih.gov/).

### Data dependent acquisitionand parallel reaction monitoring

PRM experiments were performed to confirm the expression levels of proteins obtained from quantitative proteomics. According to the results of the quantitative proteomic analysis, a list containing the mass-to-charge ratio of the unique precursor peptides of the proteins of interest was chosen for PRM analysis. A list of these peptides is shown in **Table S2**. For DDA-based experiments, peptides were analyzed in DDA acquisition mode using an online nano-flow EASY-nLC 1200 system with an analytical column (0.3 mm × 150 mm, 3 μm particle size, ChromXP, C18). Samples were eluted using solvent B (0.1% FA dissolved in 100% ACN) at a constant flow rate of 300 nl/min for 100 min with the following gradient: 2–6%, 0–1 min; 6–17%, 1–61 min; 17–23%, 61–74 min; 23–32%, 74–87 min, 32–38%, 87–90 min; 39–90%, 90–91 min; 90%, 91–100 min. A full-scan MS event was acquired between 300 and 1,800 m/z at a 60,000 resolution, and the top 20 precursor ions (200–2,000 m/z) were selected for subsequent MS/MS scans. The isolation window was set to 2.0 m/z. Fragmentation was performed using HCD with an NCE of 28% and analyzed with a resolution of 15,000 in Orbitrap. The dynamic exclusion was set at 30 s. The maximum injection times for both full MS and MS/MS were 30 ms and 50 ms, respectively. The AGC targets for both MS and MS/MS were set to 3E6 and 2E5, respectively. For the PRM experiments, the peptides were analyzed in PRM acquisition mode. MS/MS was analyzed at a resolution of 60,000 with an isolation window of 1.2 m/z. The maximum injection times for both full MS and MS/MS were 50 and 110 ms, respectively. The remaining parameters were the same as those used in the DDA-based experiments.

### Data analysis of PRM

Protein identification was performed using ProteomeDiscovery v2.3 software (Thermo Fisher Scientific). The DDA datasets were searched in the *Synechocystis* protein database using CyanoBase (http://genome.kazusa.or.jp/cyanobase) (Fujisawa et al., 2017). The parameters were set as follows: two maximum missed cleavage sites were permitted for trypsin; carbamidomethylation (Cys) was set as a fixed modification; oxidation (Met), deamidation (Asn/Gln), and acetylation (protein N-terminal) were set as variable modifications. The mass deviations of the precursor and fragment ions were set to 10 ppm and 0.02 Da, respectively. The FDR was set to <1%. For the acquired PRM data, raw and msf files were imported into Skyline (v.3.5.1.9942) (MacLean et al., 2010) to analyze peptide transitions. Peptides with low signal-to-noise ratios and/or evidence of any interference were given no further consideration and the 3–5 transitions and 3–10 most intense fragment ions were used. Peaks were manually checked for correct integration, and the transition peak areas under the curve (AUC) of the targeted peptides were obtained from the summed AUCs of each transition. The abundance of each peptide was normalized to the average abundance of each protein. The mean value of the targeted peptide abundance was used to calculate the fold change among the same protein.

## Results

### Evolutionary conservation of Clp protease family and ClpX protein

**Figure 1:**
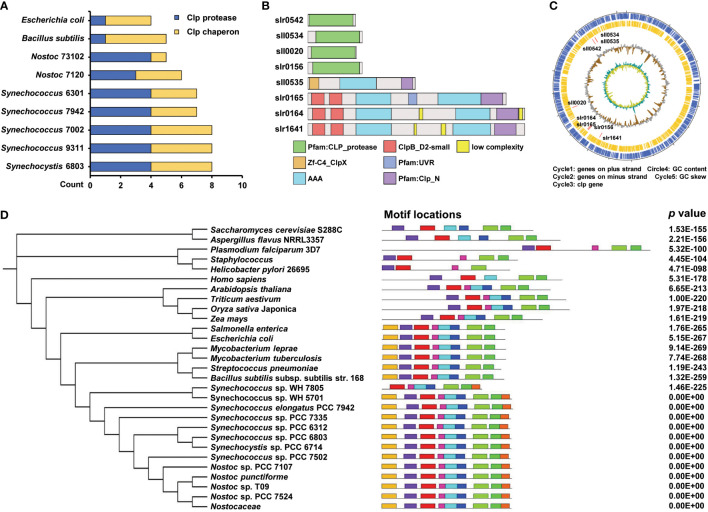
Conservativeness analysis of Clp family. **(A)** Comparing the number of *Synechocystis* sp. PCC 6803 Clp protease family homologs in different species. The blue and yellow columns indicate the count of Clp protease and Clp chaperon, respectively. **(B)** Schematic diagram of protein domains of Clp protease family from *Synechocystis* sp. PCC 6803. **(C)** The circos diagram indicates the whole *Synechocystis* sp. PCC 6803 genome. The outermost layer and inner layer denote the genes on the plus strand and minus strand in the genome, respectively. The third, fourth, and innermost layers denote the genomic position of the 8 Clp protease family members, % GC content, and GC skew, respectively. **(D)** Phylogenetic tree and motifs of ClpX protein. The tree was generated using a neighbor-joining algorithm. The conserved motif was analyzed using the MEME tool and nine different motifs were identified. Different motifs are denoted by different borders and colors, and the same color in different ClpX proteins refers to the same motif. Their combined p values are on the right side of the figure.

The Clp protease family is found in almost all bacterial species. The Clp protease system consists of two main components: Clp proteases and Clp molecular chaperones. In *Synechocystis*, the Clp protease family contains five proteolytic-like proteins (ClpP1, ClpP2, ClpP3, ClpR, and ClpP4) and four Clp molecular chaperones (ClpX, ClpB1, ClpB2, and ClpC). First, we performed a conservation analysis of *Synechocystis* Clp proteases with other species, including *Synechococcus* 7002, *Nostoc* 7120, *Synechococcus* 7942, *Synechococcus* 9311, *Nostoc* 73102, *Bacillus subtilis*, and *Escherichia coli*, using BLASTP, the similarities in percentage were shown in **Table S3**. The Clp proteases and molecular chaperones of *Synechocystis* are highly conserved across all species. The protease ClpP subunits shared 71% to 87% identity with their cyanobacterial counterparts, sharing 67.37% identity with *B. subtilis*, and 67.02% with *E. coli*. The chaperone ClpX of *Synechocystis* was found to be conserved in all other species, showing 75–82% identity with other cyanobacteria, 62.53% with *B. subtilis*, and 60.66% with *E. coli*. The results indicated that Clp proteins are highly conserved across different species. We speculate that the functions of these proteins may be conserved and essential in these species, especially in cyanobacteria. The number of homologous proteins in each species is shown in **Figure 1A**. To further characterize the structure of these Clp proteins in *Synechocystis*, we performed structural domain analysis of the Clp proteins using the SMART online website. Among these Clp proteins, the typical domain architecture of the Clp molecular chaperone consists of a specific N-terminal domain (colored red or orange) that serves as a binding site for adaptor proteins and substrates, followed by one or two characteristic conserved modules, namely AAA modules, each of which is required for ATP binding and hydrolysis (**Figure 1B**). To show the global distribution of Clp proteins in the genome of *Synechocystis*, we constructed a circular map to visualize these proteins (**Figure 1C**). Further phylogenetic analysis revealed that the ClpX protein is highly conserved among different species, especially in cyanobacteria (**Figure 1D**). To analyze the structural features of ClpX, we explored the conserved motifs of this protein using the MEME tool (http://meme-suite.org/tools/meme). As shown in **Figure 1D** and **Figure S1**, almost all prokaryotes included eight conserved motifs among different species. However, only one specific motif has been identified in cyanobacteria. We speculated that it might play an important role in fulfilling a specific regulatory function.

### Functional effects of *ClpX* in *Synechocystis*

**Figure 2:**
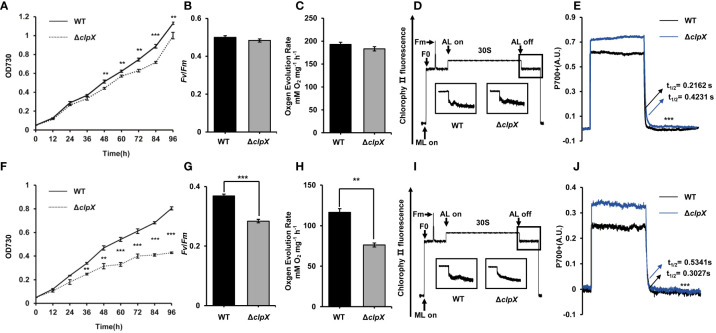
Functional effects of *clpX* in *Synechocystis.* **(A)** Growth curves of the wild type (WT) and Δ*clpX* strains under normal light (NL). **(B)** Measurement of maximum photochemistry efficiency (*Fv*/*Fm*) of the WT and Δ*clpX* strains under NL. **(C)** Measurement of oxygen evolution rates of the WT and Δ*clpX* strains under NL. **(D)** Analysis of the transient increase in chlorophyll fluorescence after the termination of AL illumination of the WT and Δ*clpX* strains under NL. **(E)** P700^+^ reduction kinetics of the WT and Δ*clpX* strains in the presence of DCMU under NL. **(F)** Growth curves of the WT and Δ*clpX* strain under high light (HL). **(G)** Measurement of maximum photochemistry efficiency (*Fv*/*Fm*) of the WT and Δ*clpX* strains under HL. **(H)** Measurement of oxygen evolution rates of the WT and Δ*clpX* strains under HL. **(I)** Analysis of the transient increase in chlorophyll fluorescence after the termination of AL illumination of the WT and Δ*clpX* strains under HL. **(J)** P700^+^ reduction kinetics of the WT and Δ*clpX* strains in the presence of DCMU under HL. ML, measuring light; AL, actinic light. Data are presented as the mean ± SD from three independent experiments. Statistical significance was determined by two-sample Student’s *t*-test (**, *p* < 0.01; ***, *p* < 0.001).

To explore the function of ClpX, we constructed a *clpX* insertion mutant (Δ*clpX*) using a homologous recombination strategy **(** **Figure S2** **)**. We measured the ability of the Δ*clpX* strain to grow photoautotrophically. As expected, the growth rate of the Δ*clpX* strain was slower than that of the wild-type (WT) strain under normal light conditions (constant illumination of 40 μmol photons m^−2^ s ^−1^) (**Figure 2A**). Besides, we observed the cell morphology and cell membrane morphology of WT and Δ*clpX* strain. No significant difference in morphology was detected between WT and Δ*clpX* strain **(** **Figures S3**, **S4** **)**. Because *Synechocystis* is a model cyanobacterium capable of oxygenic photosynthesis, we examined the photosynthetic phenotype in both the WT and Δ*clpX* strains. We found that the maximal photochemical quantum yield of PSII (*Fv*/*Fm*) and oxygen evolution rates of the Δ*clpX* strain were similar to those of the WT (**Figures 2B-C**). Interestingly, the Δ*clpX* strain exhibited a slight difference in the post-illumination chlorophyll fluorescence increase (**Figure 2D**). Considering that post-illumination increases in chlorophyll fluorescence are thought to be involved in the cyclic electron flow around photosystem I (PSI), this suggests that ClpX may affect PSI cyclic electron transport. Furthermore, P700^+^ oxidation-reduction kinetics showed that the P700^+^ oxidation-reduction rate decreased overall, and the half-life (t_1/2_) of the Δ*clpX* strain was significantly longer than that of the WT strain (**Figure 2E**). Therefore, our findings demonstrate that ClpX may play a regulatory role in PSI cyclic electron flow. As mentioned above, *clpX* interruption under normal light conditions affects growth and cyclic electron transport. We further found that the growth rate of the Δ*clpX* strain was significantly lower than that of the WT strain when cultured under high-light conditions (**Figure 2F**). Consistently, the *Fv*/*Fm*, oxygen evolution rate, post-illumination increase in chlorophyll fluorescence, and P700^+^ oxidation-reduction rate were significantly lower in the Δ*clpX* strain, compared to that of the WT (**Figure 2G-J**). To examine pigment content, the whole cell absorption spectra of WT and Δ*clpX* strain were detected, and their absorbances of all kinds of pigments were similar **(** **Figure S5** **)**. Besides, we detected the relative proportion of intracellular ROS between WT and Δ*clpX* and their lipid peroxidation levels. Obvious differences were observed between WT and Δ*clpX* strain in these experiments, suggesting the Δ*clpX* strain could be under oxidative stress **(** **Figure S6** **)**. Collectively, these observations demonstrate that ClpX plays an important role in cell growth and photosynthesis in *Synechocystis*.

### Identification of ClpX-regulated proteins in *Synechocystis*

**Figure 3:**
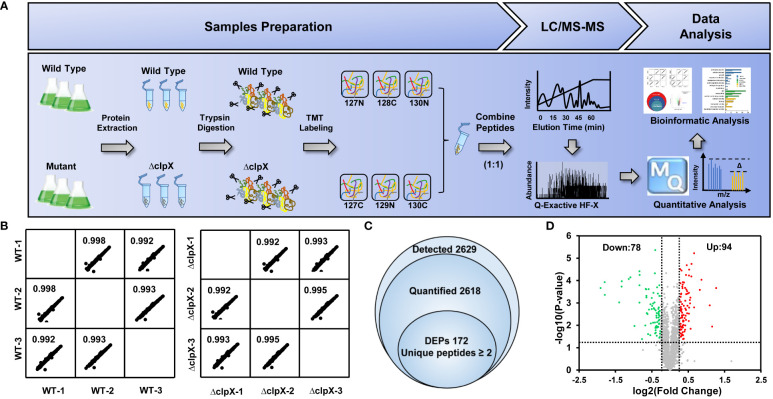
Workflow and quantitative proteomics data. **(A)** Workflow for TMT labeling quantitative proteomic strategy. **(B)** Pairwise correlation of peak area of identified proteins between three biological replicates. **(C)** Venn diagram showing the overlap of the number of detected proteins quantified proteins, and differentially expressed proteins (DEPs). **(D)** Volcano plots showing *p*-values (–log_10_) versus the fold change (log_2_) of DEPs. Proteins with *p* < 0.05 and fold change > 1.2 or < 0.83 are considered to be differentially expressed. Upregulated and downregulated proteins are marked as red and green dots, respectively.

To explore the potential regulatory mechanism of the ClpX protein in *Synechocystis*, a tandem mass tag (TMT)-labeled quantitative proteomic strategy was used to identify dysregulated proteins upon the interruption of *clpX* (**Figure 3A**). Pearson’s correlation coefficients were calculated based on the log_2_-transformed protein intensities among the three biological replicates of the WT and *ΔclpX* groups to assess the reproducibility of the TMT-based quantitative proteomic data. The strong correlations with corresponding coefficients *r* > 0.99 indicated high repeatability among the three replicates **(** **Figure 3B** **)**. The high-quality proteomic data obtained enabled us to identify 2,629 proteins **(** **Table S4** **)**, accounting for approximately 75% of the predicted *Synechocystis* proteins. Among these, 2,618 proteins were quantifiable using the stringent filtering criteria described in the Materials and Methods section. A total of 172 proteins were differentially expressed between the WT and ΔclpX strains according to the standard of p-value<0.05 and |log2FC|≥1.20 **(** **Figure 3C** and **Table S5** **)**. Among these DEPs, 94 proteins showed a fold change ≥ 1.20 and 72 proteins showed a fold change ≤ 0.83. Volcano plots, in which the fold change (log_2_) of DEPs is plotted against the corresponding *p*-value, are shown in **Figure 3D**. These DEPs will provide novel candidates for future studies that will allow assessment of their physiological roles and significance in ClpX-regulated processes.

### Functional characterization of ClpX-regulated proteins

**Figure 4:**
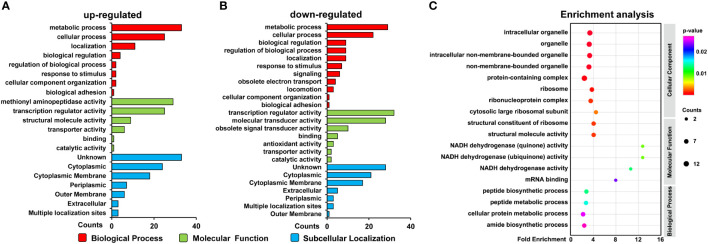
Functional analysis of differentially expressed proteins (DEPs). **(A)** GO classification of the upregulated proteins and **(B)** downregulated proteins. **(C)** GO enrichment of the DEPs. Circle size indicates the number of enriched proteins. Color saturation represents the significance level.

To better understand the biological function of ClpX-regulated proteins, all identified DEPs were annotated according to the gene ontology (GO) system using Blast2GO software **(** **Table S6** **)**. The annotation results obtained from the biological processes demonstrated that most of the DEPs were involved in metabolic and cellular processes, localization, biological regulation, and response to a stimulus. Moreover, a group of down-regulated proteins was found to be involved in signaling and obsolete electron transport. From this perspective, our observations support the previous conclusion that ClpX-regulated proteins may play an important role in metabolism regulatory functions and stress responses (**Figures 4A-B**) (Sokolenko et al., 2002). Regarding molecular functions, DEPs were widely distributed in transcription regulator, transducer, and catalytic activity, as well as binding. Some downregulated proteins were annotated as having antioxidant activity, indicating the importance of ClpX in the stress response (**Figures 4A-B**). Although the subcellular localization of proteins will provide novel insight into their biological function, the localization of the majority of the DEPs was not predicted (unknown), according to the subcellular localization analyzed by PSORTb **(** **Table S7** **)**. In addition, a large number of DEPs were assigned to the cytoplasm, where a series of biological processes such as carbon, nitrogen, and amino acid metabolism, and protein degradation occur. These results were in line with the results of the biological process annotation that considerable DEPs were classified as being involved in metabolic and cellular processes, and biological regulation. In addition, some DEPs were located in the outer and cytoplasmic membrane, suggesting that these DEPs may be related to stimuli, signal transducer activity, transporter activity, and obsolete electron transport (**Figures 4A-B**).

GO enrichment analysis was performed to elucidate the biological functions of the ClpX-regulated proteins **(** **Table S8** **)**. As depicted in **Figures 4C**, our data showed that ClpX-regulated proteins were significantly enriched in the peptide biosynthetic process and peptide and cellular protein metabolic processes, based on biological process enrichment. According to the results of the molecular function enrichment analysis, we found that the DEPs were mostly enriched for binding and enzymatic activities, such as mRNA binding and NADH dehydrogenase activity. Within the GO cellular component categories, a large proportion of DEPs were associated with ribosomes, ribonucleoprotein complexes, cytosolic large ribosomal subunits, and structural constituents of ribosomes. Our functional annotation analysis indicated widespread roles of ClpX in fulfilling the delicate regulatory function in *Synechocystis*, similar to that in other organisms (Claunch et al., 2018; Lo et al., 2020; Kirsch et al., 2020).

### Functional interaction networks of ClpX-regulated proteins

To further explore the biological roles of the identified DEPs, an overall protein-protein interaction network (PPI) was constructed using the STRING database. The network incorporated 94 nodes and 197 edges and was visualized using Cytoscape (**Figure S7** and **Table S9**). Each edge was examined using a score as the edge weight to quantify interaction confidence. Usually, the average node degree is used to represent the average number of edges per node in a PPI network, and a higher value for the degree indicates a highly connected network and is likely to be more robust. The average node degree of 3.12 implicated more edges connecting to nodes in our data. Moreover, a small *p*-value (3.85 × 10^-08^) of PPI enrichment was obtained in our network, suggesting that the observed degree of edges was significant, and the identified DEPs were functionally connected. Based on this network, we characterized protein complexes of DEPs, and six highly interconnected clusters were observed according to the Markov cluster algorithm (MCL). In line with our GO annotation results, the top cluster (cluster I), clusters III, IV, and VI consisted of DEPs related to transport-related, signal transduction mechanism, anti-sigma factor antagonist domain, and phosphate transport, implying that these DEPs may play functional roles in transporter activity and stress response. Cluster V consisted of metabolism-associated proteins, such as Nodb homology and xylose isomerase-like domain complex. These findings suggest that ClpX-regulated proteins are involved in diverse cellular processes in *Synechocystis.*

### ClpX-regulated proteins involved in metabolism

Accumulating evidence has revealed that ClpX can recognize the amino acid sequences of substrates, serving as tethering or degradation tags, for further protease-mediated protein degradation (Zhang and Zuber, 2007). Based on a previous study (Kirsch et al., 2020) and the results of our functional annotation, we anticipated that *clpX* interruption would disturb cellular metabolic processes in *Synechocystis*. Consistent with this notion, DEPs were found to be involved in metabolic pathways, ATP-binding cassette (ABC) transporters, signal transduction, and defense mechanisms **(** **Table S10A** **)**. As shown in **Figure S8**, **S9A**, *clpX* interruption led to the downregulation of key enzymes involved in carbon metabolism, including hydrolysis of glycogen (glgX, encoded by slr1857) and the glycolytic pathway (pfkA2, encoded by sll0745; and yibo, encoded by slr1945). In addition, *clpX* interruption led to significantly decreased expression of proteins involved in nitrogen metabolisms, such as the cytochrome b subunit of nitric oxide reductase norB (sll0450) and glutamate–ammonia ligase glnN (slr0288). Interestingly, the proteins involved in purine and pyrimidine metabolism, such as ‘*de novo*’ UMP biosynthetic process protein (sll0744), were also downregulated, which was in line with a previous study (Kirsch et al., 2021). The interruption of *clpX* affects the two-component signal transduction system and ABC transporters of phosphate, iron, manganese, phospholipid, and sulfate. Consistently, all phosphate transporter-associated proteins, including sphX (sll0679), pstS (sll0680), pstB1 (sll0683), pstB2 (sll0684), pstC (sll0681), pstC (slr1248), pstS (slr1247), and ziaA (slr0798) were significantly downregulated, whereas the iron(III) transporter hitB (slr0327), manganese transporter mntC (sll1598), phospholipid transporter ycf22 (sll1002), and sulfate transporter bicA (sll0834) exhibited significant upregulation, indicating the presence of different regulatory roles of ClpX, similar to the results from other organisms. Moreover, two-component system proteins, such as two-component sensor histidine kinase (sll1590), two-component hybrid sensor and regulator (sll1296), and two-component system response regulator (sll1330), were also significantly upregulated, suggesting that ClpX may play an important role in stress response. Notably, because the Clp protease complex can degrade most ribosomal proteins (Kuroda et al., 2001; Kuroda, 2006), the interruption of *clpX* may consequently give rise to the upregulation of ribosomal constituent proteins (mainly the 50S large subunit), including rplB (sll1802), rplX (sll1807), rpsK (sll1817), rplT (sll0767), rpsL (sll1096), and rpmI (ssl1426). Furthermore, many proteases, such as hhoA (sll1679), ymxG (slr1331), htrA (slr1204), methionine aminopeptidase (sll0555), and putative carboxypeptidase (sll0777), were significantly upregulated. As mentioned above, ClpX functions as a chaperone for proteases, assisting in the protein degradation process. Therefore, the interruption of *clpX* may affect the proteolytic function of proteases, and some compensatory effects may be stimulated.

Among the 172 DEPs, 89 were annotated as hypothetical or unknown proteins in the *Synechocystis* database. Next, we performed functional annotation for these hypothetical or unknown proteins using BLASTP homology searches and CD-search for conserved domain annotations **(** **Table S10B** **)**. Notably, more than half of these proteins can be annotated and assigned to metabolic pathways, ABC transporters, two-component signal transduction, regulation of gene expression, protein degradation, and defense mechanisms (**Figures S8**, **S9B**). These findings provide new candidates for future functional studies of ClpX.

### Validation of the DEPs by parallel reaction monitoring (PRM) analysis

**Figure 5:**
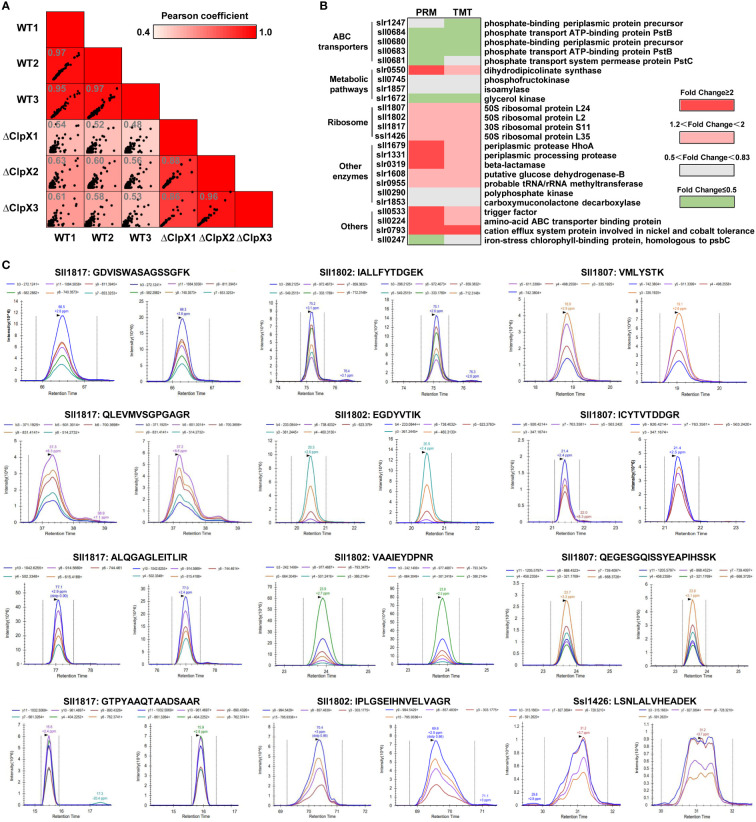
Validation of DEPs using Parallel Reaction Monitoring (PRM) analysis. **(A)** Pairwise correlation of peak area of transitions for the selected DEPs between three biological replicates. **(B)** Heatmap showing the expression levels of the DEPs selected for validation by PRM. **(C)** Chromatograms represent the fragment ion extracted-ion chromatograms (XICs) for the representative peptides from the WT and *ΔClpX* strains.

To independently verify the changes in the abundance of DEPs measured in our TMT-labeled quantitative proteomics experiments, PRM analysis was performed to further confirm twenty-four of the identified DEPs **(** **Table S11** **)**. These validation experiments were performed on the same batch of extracted peptide samples used for quantitative proteomics experiments. All data were imported into Skyline software to further check the peak shape and retention time of each peptide segment. To evaluate the correlation between PRM-based protein expression and TMT-labeled quantitative proteomics data, we performed a Pearson correlation analysis based on the log_2_-transformed abundance of transitions for the 24 selected proteins among the biological replicates. According to the scatter plot, the strong linear correlation observed indicates a high level of reproducibility among the replicates **(** **Figure 5A** **)**. We then constructed a heatmap based on the 24 selected proteins to analyze the consistency of expression levels between PRM-based proteins and TMT-labeled quantitative proteins. As shown in **Figure 5B**, the quantified results obtained from PRM were mostly in agreement with those of TMT-labeled quantitative proteomics. The identified DEPs were grouped into five categories according to their specific functions. ClpX-regulated proteins involved in the regulation of gene expression and protein degradation, as well as defense mechanisms, were significantly upregulated. The selected proteins in metabolic pathways were downregulated, except for dihydrodipicolinate synthase (slr0550). The remaining proteins, including ABC transporter-binding protein and cation efflux system protein involved in nickel and cobalt tolerance, were upregulated in the *ΔclpX* strain, whereas the other proteins in ABC transporters and signal transduction were mostly down-regulated. The high consistency indicated that our PRM validation assay was reliable for measuring relative protein expression levels. The extracted transitions of the representative peptides from 50S ribosomal protein L2 (sll1802), 50S ribosomal protein L24 (sll1807), 50S ribosomal protein L35 (ssl1426), and 30S ribosomal protein S11 (sll1817) are shown in **Figure 5C**.

## Discussion

ClpX functions as a chaperone of the ATP-dependent protease ClpP, forming ClpXP proteolytic complexes, which are essential for maintaining proteostasis by disposing of damaged or unneeded proteins, as well as for the conditional degradation of functional proteins in response to external or internal signals (Sauer et al., 2004). However, the molecular details of this process remain poorly understood.

To globally search for ClpX-regulated proteins in *Synechocystis*, we compared the protein profiles of the WT and Δ*clpX* strains using a quantitative proteomic strategy. A total of 172 DEPs were identified, and the differential expression levels of 24 proteins were confirmed by PRM analysis. Bioinformatics analysis suggested that these DEPs were enriched in a variety of biological regulatory pathways, including glycolysis, amino acid biosynthesis, nitrogen assimilation, photosynthetic electron transport, ABC transporters, and two-component signal transduction. Therefore, we provided a proteome-wide view of the regulatory networks of ClpX in this model cyanobacterium.

In this study, we demonstrated that ClpX plays an essential role in growth. It is reported that ClpXP proteolytic complex plays a role in cell division by modulating the level of FtsZ through degradation, and may degrade multiple cell division proteins, thereby modulating the balance of the components required for division (Camberg et al., 2011). Moreover, ClpX is required to relieve a clock-induced cell division checkpoint (Cohen et al., 2018). Furthermore, NOA1, which is essential for mitochondrial protein synthesis, oxidative phosphorylation, and ATP production, is one of the substrates of the ClpXP proteolytic complex (Al-Furoukh et al., 2014). Overall, these reports were consistent with our experimental results, and ClpX may influence the growth of cyanobacteria by affecting cell division and metabolism.

**Figure 6:**
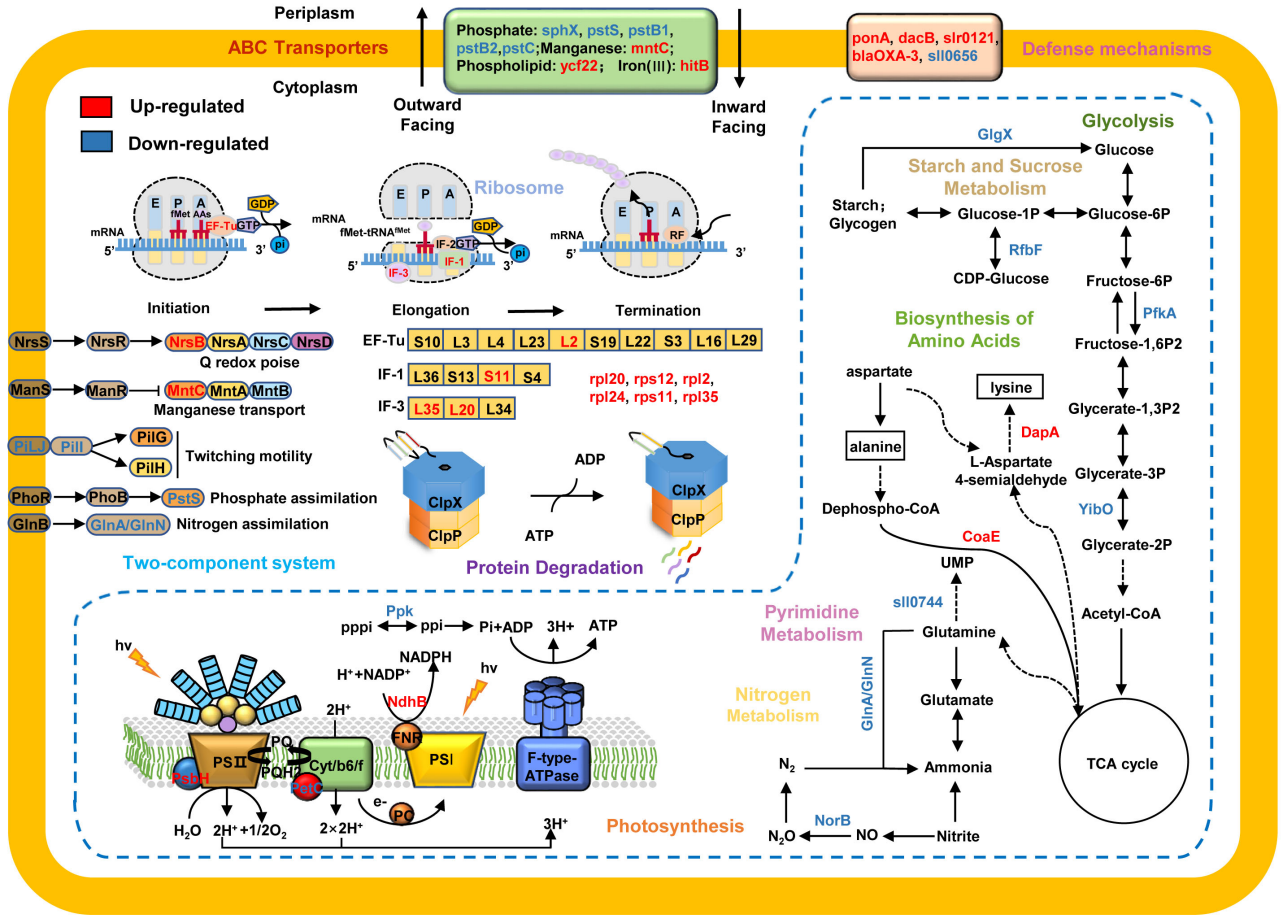
** **A proposed model showing the regulatory networks of ClpX in *Synechocystis* sp. PCC 6803. The upregulated proteins are highlighted in red and downregulated proteins are in blue. Arrows represent state transitions or metabolite fluxes.

Based on previous reports (Rowland et al., 2011) and our results, we mapped ClpX-regulated proteins to KEGG pathways and constructed a hypothetical model to depict the potential regulatory mechanisms of ClpX in *Synechocystis* (**Figure 6**). Cyanobacteria have an intricate light-harvesting apparatus that captures light and synthesizes ATP and NADPH by driving photosynthetic electron transport pathways (Mullineaux, 2014). As it’s an essential step for the degradation of photodamaged proteins in photosynthetic organisms (Stanne et al., 2009), several dysregulated proteins associated with the electron transport chain, including photosystem II PsbH protein (ssl2598), cytochrome b6f complex petC (sll1182), and ferredoxin (ssl2559), were identified in the Δ*clpX* strain, owing to the existence of reduced photosynthetic performance. In addition, our experimental results demonstrated that the interruption of *clpX* decreased the P700^+^ oxidation-reduction rate in the presence of DCMU and that cyclic electron transport was dominant at the same time. The interruption of *clpX* also influenced the increase in transient chlorophyll fluorescence, which is involved in PSI cyclic electron transport. Thus, our data suggest that ClpX may influence cyclic electron transport by regulating the expression of these photosynthesis-related proteins.

Given the important roles of ClpX in stress responses, such as temperature (LaBreck et al., 2017; Roy et al., 2019), pH (Roy et al., 2019), and Fe^2+^ stresses (Bennett et al., 2018), we anticipated that ClpX may also have functions in the high light response in cyanobacteria. As expected, the Δ*clpX* strain exhibited slow growth and photosynthetic electron transport rates under high light conditions (**Figure 2**). It is noteworthy that many proteins associated with ABC transporters and two-component signal transduction systems were dysregulated after *clpX* interruption, such as phosphate assimilation, nitrogen availability, and manganese starvation (**Figure 6**). The two-component signal transduction system represents a crucial means of sensing and responding to environmental changes both intra- and extracellularly in bacteria (Koretke et al., 2000). The sensor histidine kinase can respond to intra- or extracellular signals by catalyzing the phosphorylation of related response regulators, which are then capable of adjusting gene expression or cellular physiology to cope with the changes that occur in its environment (Storz and Hengge, 2010). ABC transporters are membrane proteins that couple the transport of diverse substrates across cellular membranes to ATP hydrolysis (Hollenstein et al., 2007), which plays an important role in environmental adaptation. Increasing evidence suggests that ABC transporters and signal transduction systems often work together to respond to environmental change (Dintner et al., 2011; Dintner et al., 2014). In this study, we identified several DEPs involved in the Pst system, which is an ATP-dependent ABC transporter-type system that includes phosphate-binding proteins and transmembrane protein units (Rao and Torriani, 1990). For instance, several Pst system members were downregulated after *clpX* interruption, including sll0680 (pstS), sll0681 (pstC), sll0683 (pstB1), slr1248 (pstC), and slr1247 (pstS). PstS is a phosphate-binding periplasmic protein commonly found in cyanobacteria, PstA and PstC are transmembrane subunits that form a channel in the inner membrane, and PstB is a membrane protein that contains an ATP-binding domain (Su et al., 2007; Jin et al., 2021). PstA, PstB, and PstC constitute the ABC-type Pst transporter system. PstS can bind to Pi and transport it through the Pst transporter system by hydrolysis of ATP and interacts with the Pst transporter system to transmit the signal to the sensor kinase SphS (Rao and Torriani, 1990; Tiwari et al., 2015). Likewise, phosphate availability sensing is regulated by a two-component regulatory system, SphS-SphR, in cyanobacteria, which is orthologous to PhoB-PhoR in *E. coli* (Hirani et al., 2001; Suzuki et al., 2004). In this system, specific environmental signals are sensed by membrane-bound histidine kinases, activating response regulators to regulate the expression of target genes and finally mediate specific cellular responses against the stimulus (Mascher et al., 2006). Based on these data, we suggest that ClpX may affect the high-light adaptation of *Synechocystis* through the regulation of a two-component signal transduction system and ABC transporters.

In this study, many ribosomal proteins were found to be dysregulated in the Δ*clpX* strain, suggesting that ClpX may affect global protein abundance by regulating the expression of ribosome-associated proteins (**Figure 6**). Ribosomes are macromolecular machines responsible for the process of translation, and encode mRNAs into polypeptide chains with high speed and accuracy (Green and Noller, 1997). Ribosomes fold polypeptides during their synthesis to decrease the risk of protein misfolding and aggregation, contributing to the maintenance of proteostasis (Cassaignau et al., 2020). Although several intricate mechanisms ensure efficient and precise protein synthesis, some mistakes inevitably occur during protein synthesis (Frischmeyer et al., 2002; Haebel et al., 2004). Misfolded or partially folded proteins often aggregate and/or interact inappropriately with other components, leading to the impairment of cell viability and eventually cell death (Mishra and Grover, 2016). Specific proteases, including ClpAP and ClpXP, which can target specific degradation signals, are recruited to degrade these aberrant proteins (Gottesman et al., 1998; Song and Eck, 2003). Several ribosomal proteins can be captured by the ClpXP complex in *E. coli* (Flynn et al., 2003). The isolated ribosomal complex also contains the ClpXP complex in *E. coli*, HepG2, and human cell lines (Fux et al., 2019). Thus, our data suggest that ClpX may play a biological role by targeting ribosome-associated proteins in *Synechocystis.*

A large number of hypothetical or unknown proteins have been identified as dysregulated proteins in response to *clpX* interruption. Based on our re-annotation results, many hypothetical proteins were classified as clustered regularly interspaced short palindromic repeat (CRISPR)-associated proteins **(** **Table S7** **)**. It has been reported that *clpP* deficiency increases the expression of CRISPR-associated genes in *Streptococcus* mutant*s* (Chattoraj et al., 2010). An increasing number of studies have pointed to the direct links between CRISPR-associated proteins and the regulation of a range of stress-related phenomena (Louwen et al., 2014). For the first time, we found that *clpX* interruption may lead to the dysregulation of CRISPR-associated proteins in *Synechocystis*. This study provides new candidates for future functional studies of ClpX and novel insights into the mechanisms of protein homeostasis in cyanobacteria.

In conclusion, we established a ClpX regulatory network in *Synechocystis* based on the results of proteomic and functional studies. The identified comprehensive catalog of proteins provides a valuable resource for further mechanistic investigations of protein quality control systems in cyanobacteria.

## Data availability statement

The original contributions presented in the study are publicly available. This data can be found here: iProX, IPX0004691000.

## Author contributions

MY and FG: conceptualization. YZ, YW, WW, MW, and SJ: methodology. YZ, YW, and MY: investigation and writing-original draft preparation. WW, MW, and SJ: data curation. MY, FG: writing-review and editing. WW, MW, SJ: visualization. WW and FG: funding acquisition. All authors have read and agreed to the published version of the manuscript.

## Funding

This work was supported by the National Key Research and Development Program of China (2020YFA0907400), the National Natural Science Foundation of China (grant no. 31870756), the Chinese Academy of Sciences Grant QYZDY-SSW-SMC004, the China Postdoctoral Science Foundation to W.W. (2021M703432), and the State Key Laboratory of Freshwater Ecology and Biotechnology to W.W. (2022FB03).

## Acknowledgments

We thank the Analysis and Testing Center of Institute of Hydrobiology and Micrometer Biotech Company (Hangzhou, China) for their helps in proteomic experiments.

## Conflict of interest

The authors declare that the research was conducted in the absence of any commercial or financial relationships that could be construed as a potential conflict of interest.

## Publisher’s note

All claims expressed in this article are solely those of the authors and do not necessarily represent those of their affiliated organizations, or those of the publisher, the editors and the reviewers. Any product that may be evaluated in this article, or claim that may be made by its manufacturer, is not guaranteed or endorsed by the publisher.
