# Supplementary material for: Proteomic analysis of the regulatory networks of ClpX in a model cyanobacterium Synechocystis sp. PCC 6803
Source: Front Plant Sci. 2022 Sep 29;13:994056. doi: 10.3389/fpls.2022.994056 (PMC9560874; doi:10.3389/fpls.2022.994056)
Supplement: Supplementary file 1 [file Image_1.pdf]

Supplementary Figures

Proteomic analysis of the regulatory networks of ClpX in a model cyanobacterium *Synechocystis* sp. PCC 6803

Yumeng Zhang[1,2,3], Yaqi Wang[1,2,3], Wei Wei[1,2,3], Min Wang[4], Shuzhao Jia[4], Mingkun Yang[1,2,3*], Feng Ge[1,2,3*]

[1]State Key Laboratory of Freshwater Ecology and Biotechnology, Institute of Hydrobiology, Chinese Academy of Sciences, Wuhan 430072, China

[2]Key Laboratory of Algal Biology, Institute of Hydrobiology, Chinese Academy of Sciences, Wuhan 430072, China

[3] College of Advanced Agricultural Sciences, University of Chinese Academy of Sciences, Beijing 100049, China

[4]The Analysis and Testing Center, Institute of Hydrobiology, Chinese Academy of Sciences, Wuhan 430072, China

*To whom correspondence should be addressed: Prof. Feng Ge, E-mail: gefeng@ihb.ac.cn. Phone/Fax: +86-27-68780500. Dr. Mingkun Yang, E-mail: yangmingkun@ihb.ac.cn. Phone/Fax: +86-27-68780730.

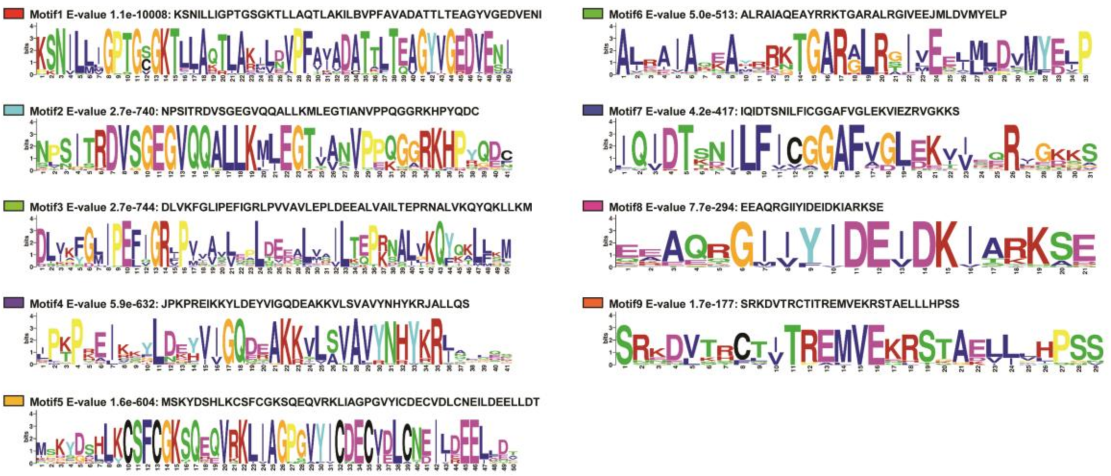

Fig. S1. The motif amino acid sequences and their corresponding E-values.

.

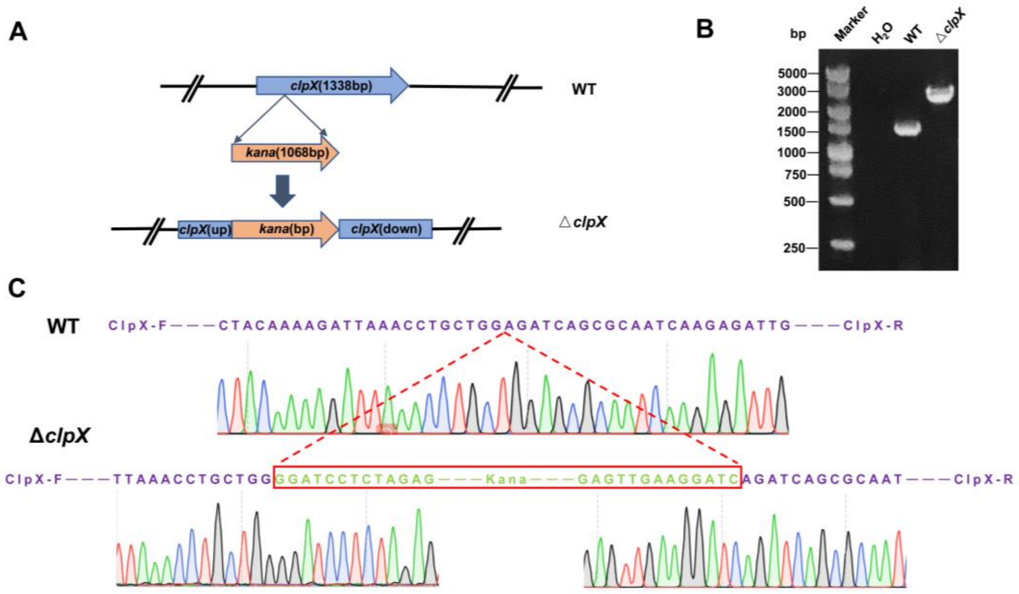

Fig. S2. The construction of *clpX* insertional mutant strain. **(A)** The schematic shows the construction of *clpX* insertional mutant strain by homologous recombination. Verification of the *clpX* insertional mutant strain by **(B)** PCR and **(C)** sequencing.

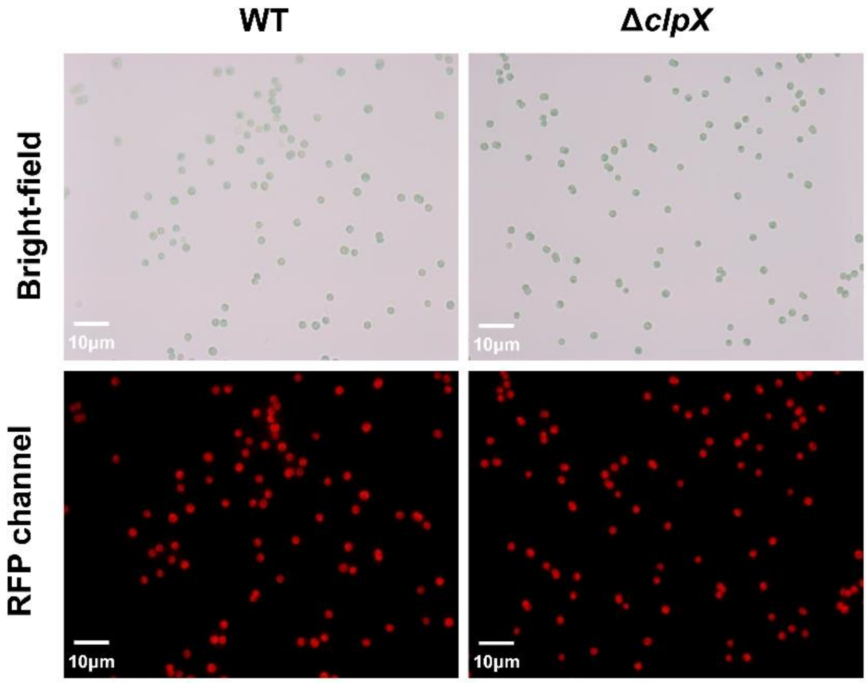

Fig. S3. Cell morphology of *Synechocystis* cells. Bright-field and fluorescent images in the RFP channel were used to determine cell morphology. Images were recorded using a fluorescent microscope (Olympus, BX53, Japan) at a magnification of 100×.

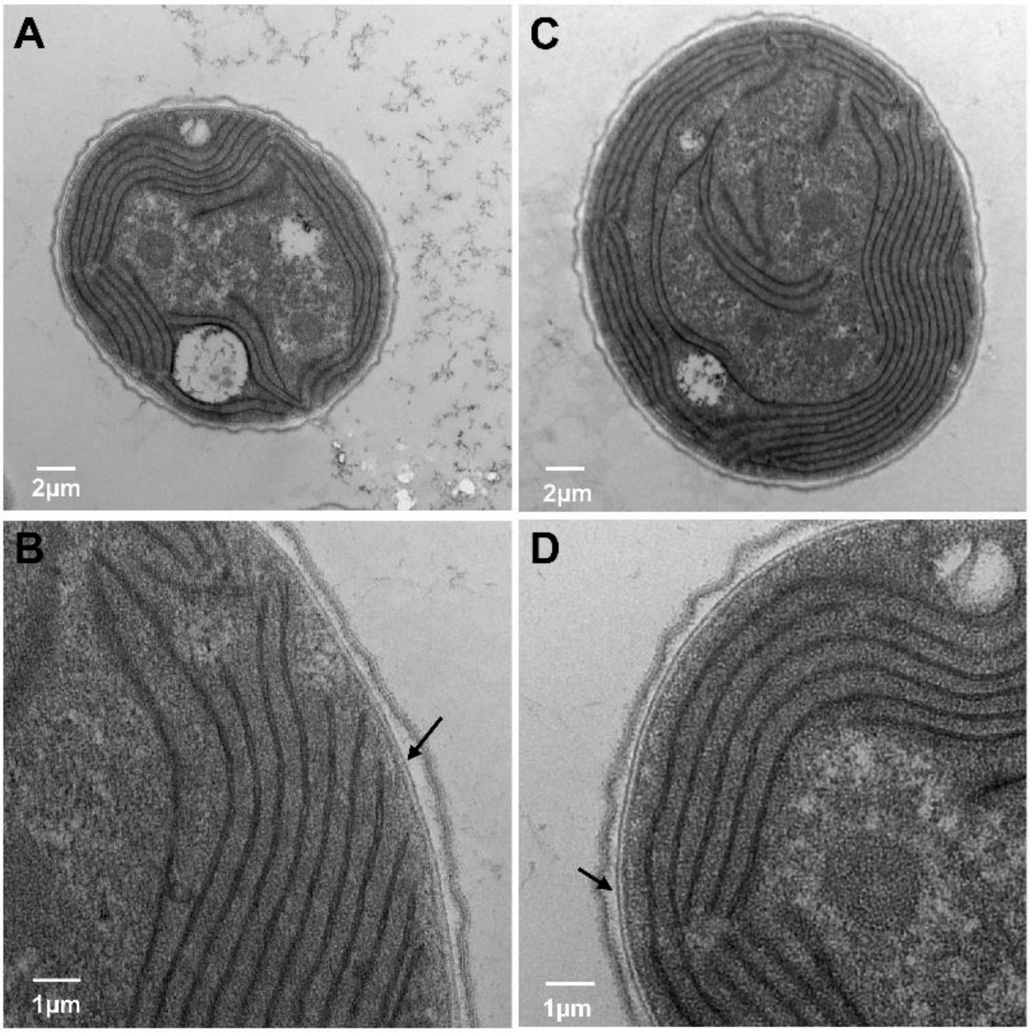

Fig. S4. Transmission electron microscopy of the *Synechocystis* cells. Stained ultrathin sections of WT **(A-B)** and Δ*clpX* **(C-D)**. The black arrow points at the cell membrane. Images were recorded using the TEM system (Hitachi, Japan) at a magnification of 10.000 × and 25.000 ×.

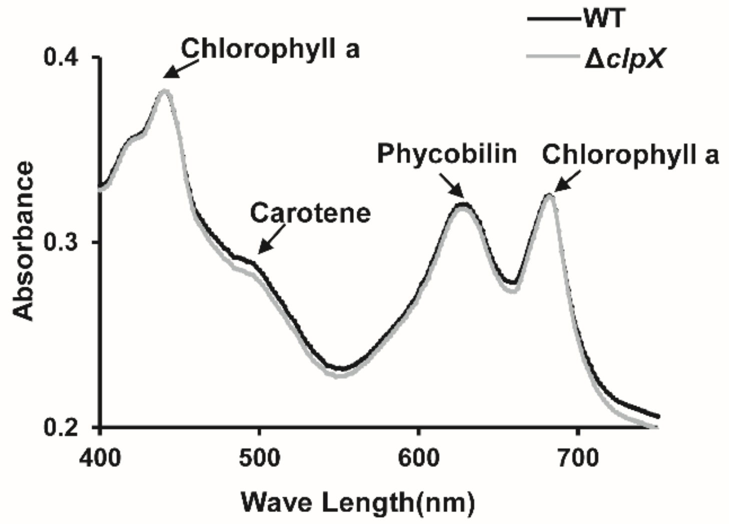

Fig. S5. The whole cell absorption spectra of WT and Δ*clpX*. Each spectrum is the average of three measurements.

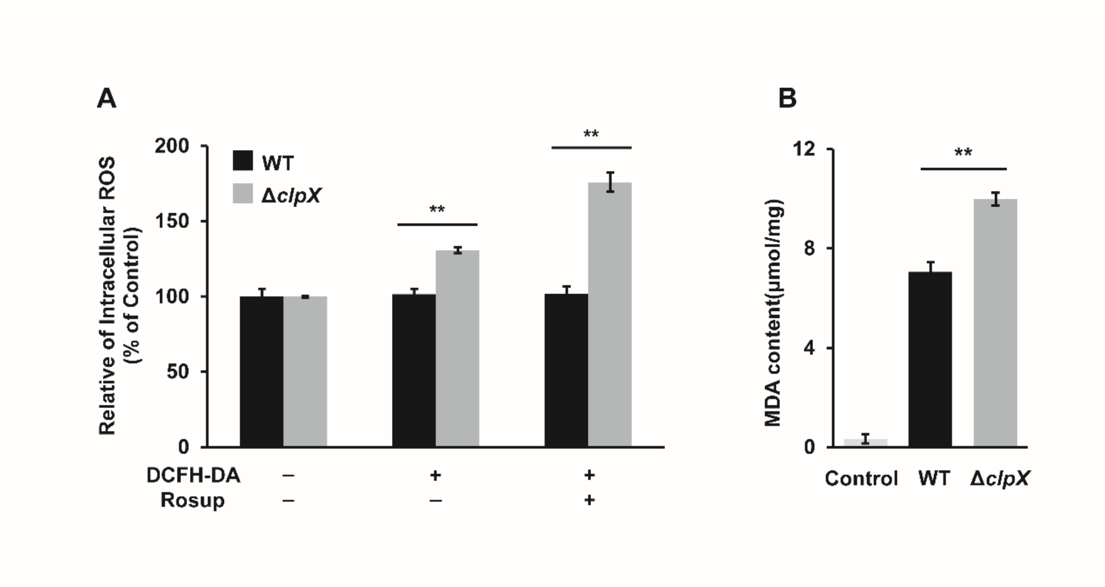

Fig. S6. The estimation of oxidative stress levels in WT and Δ*clpX* strains. **(A)** Relative quantitative analysis of DCF fluorescence intensity in WT and Δ*clpX* strains. [Relative of intracellular ROS = [(fluorescence intensity of treatment group) / (fluorescence intensity of control group) × 100%]. **(B)** The measurement of the lipid peroxidation levels in WT and Δ*clpX* strains. ROS, reactive oxygen species; MDA, malondialdehyde.

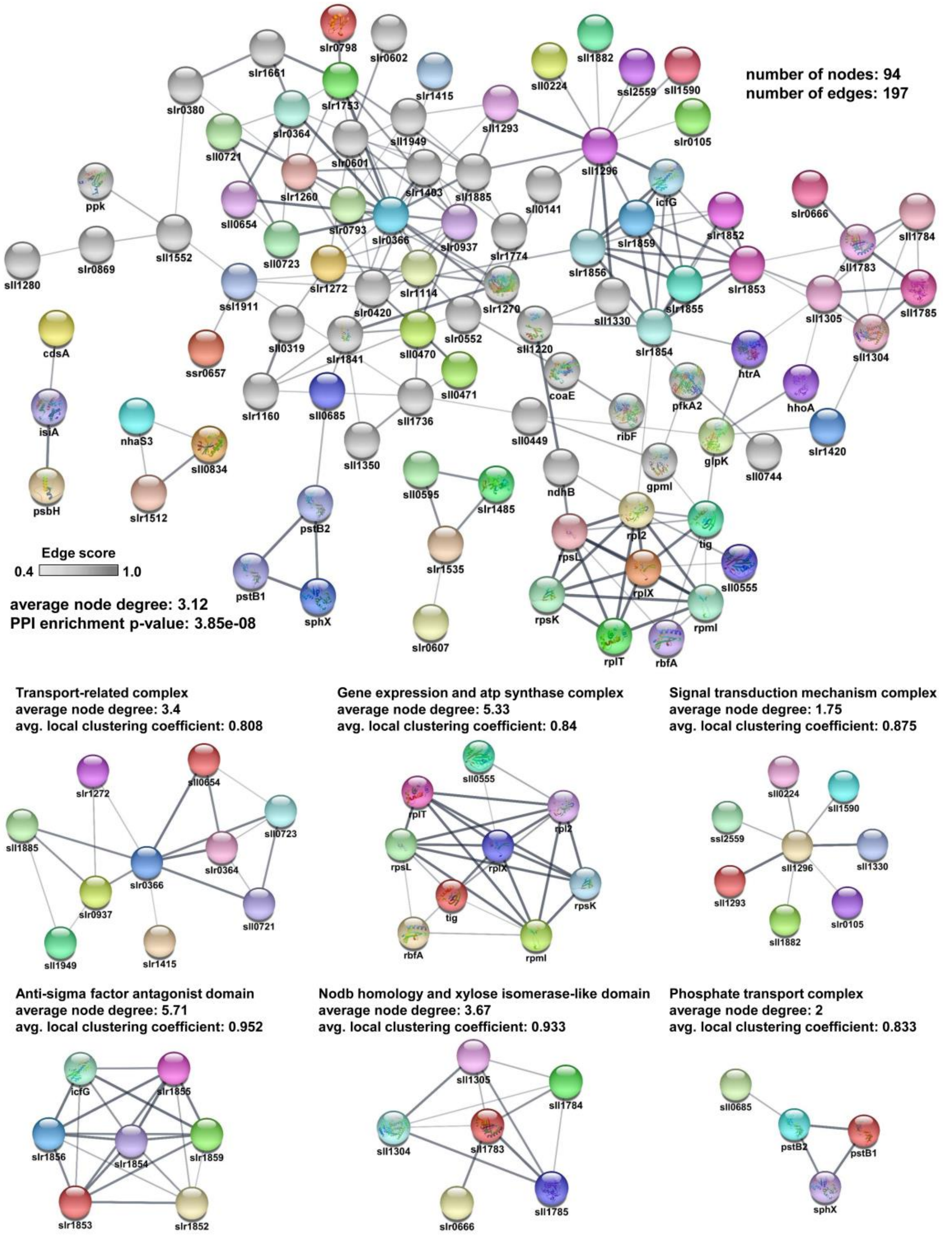

Fig. S7. The protein-protein interaction (PPI) network analysis of differentially expressed proteins (DEPs) using STRING database. The 172 DEPs were input into the STRING database for PPI network analysis, and achieved a PPI network of 3.12 average nodes, with PPI enrichment p value = 3.85 × 10^{-08}. The six primary clusters of subnetworks were analyzed by MCL clusters in STRING.

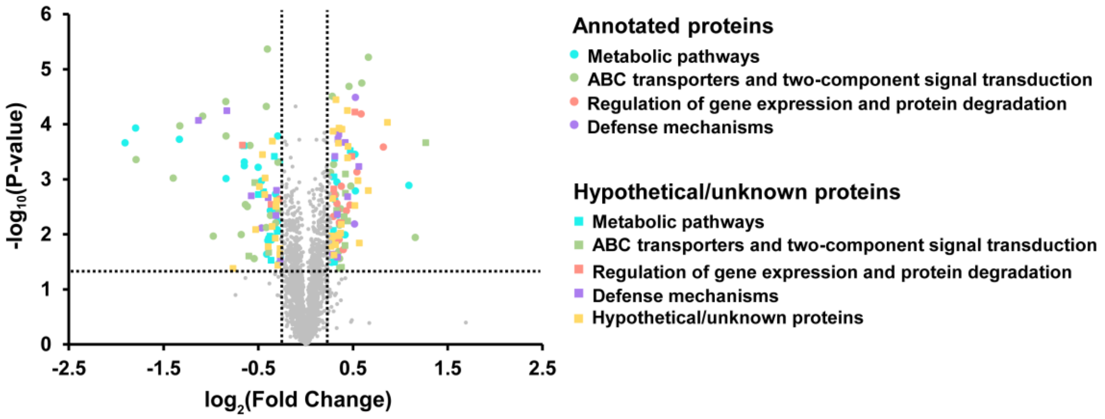

Fig. S8. Comparison of the protein profiles of wild-type and Δ*clpX* strains using a quantitative proteomic strategy. Volcano plots showing p values ($-\log_{10}$) versus the fold change ($\log_2$) of DEPs. Proteins with $p < 0.05$ and fold change > 1.2 or < 0.83 are considered to be differentially expressed. Proteins with differential expression were annotated according to their functions and highlighted in different colors.

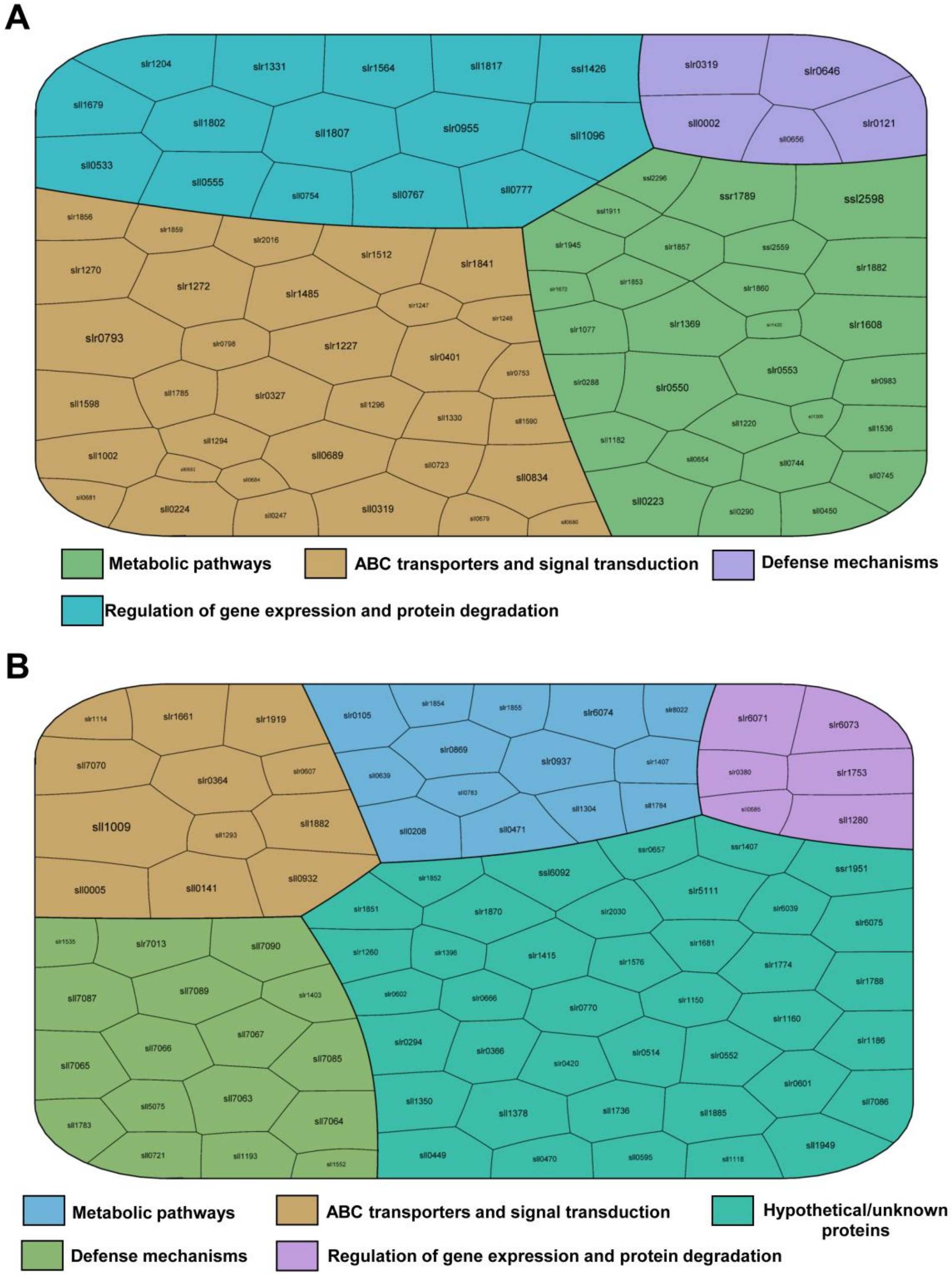

Fig. S9. The Voronoi treemap visualizes functionally organized quantitative information as area of hierarchically organized information on abundance of **(A)** annotated proteins and **(B)** hypothetical or unknown proteins. Proteins were clustered according to their functional categories as areas of different colors.
